# Constitutive Models for the Prediction of the Hot Deformation Behavior of the 10%Cr Steel Alloy

**DOI:** 10.3390/ma12182873

**Published:** 2019-09-05

**Authors:** Abdallah Shokry, Samer Gowid, Ghias Kharmanda, Elsadig Mahdi

**Affiliations:** 1 Department of Mechanical Engineering, Fayoum University, Fayoum 63514, Egypt; 2 Department of Mechanical and Industrial Engineering, College of Engineering, Qatar University, Doha 2713, Qatar (S.G.) (E.M.); 3 Mechanics Laboratory of Normandy, 76131 INSA Rouen, France

**Keywords:** 10%Cr steel alloy, hot deformation, Johnson–Cook model, strain-compensated Arrhenius model, artificial neural network

## Abstract

The aim of this paper is to establish a reliable model that provides the best fit to the specific behavior of the flow stresses of the 10%Cr steel alloy at the time of hot deformation. Modified Johnson–Cook and strain-compensated Arrhenius-type (phenomenological models), in addition to two Artificial Neural Network (ANN) models were established with the view toward investigating their stress prediction performances. The ANN models were trained using Scaled Conjugate Gradient (SCG) and Levenberg–Marquardt (LM) algorithms. The prediction accuracy of the established models was evaluated using the following well-known statistical parameters: (a) correlation coefficient (R), (b) Average Absolute Relative Error (AARE), (c) Root Mean Squared Error (RMSE), and Relative Error (RE). The results showed that both of the modified Johnson–Cook and strain-compensated Arrhenius models could not competently predict the flow behavior. On the contrary, the results indicated that the two proposed ANN models precisely predicted the flow stress values and that the LM-trained ANN provided a superior performance over the SCG-trained model, as it yielded an RMSE of as low as 0.441 MPa.

## 1. Introduction

Among the improved low alloy steels, the 10 wt.% Cr steel, X12CrMoWVNbN.10.1.1 (referred to as X12), has been widely used in high-temperature applications such as boiler and turbine components of power plants [1,2,3]. In addition to the low cost of the X12 alloy, it has superior properties at elevated temperatures, in particular creep resistance, durability, and corrosion resistance [4,5]. Therefore, considering the hot deformation behavior of the X12 alloy is essential to be able to design and analyze components that are subjected to high temperature applications.

Finite element simulations have been extensively utilized for analyzing and optimizing metal forming processes like rolling and extrusion [6,7,8]. Constitutive models that express the flow behavior of materials at different loading conditions are essential as they are utilized as an input code for finite element models. Consequently, the precision of the constitutive models has a great impact on the accuracy of the obtained results of simulation. The Johnson–Cook (JC) model [9,10] is considered as one of the significant phenomenological models. The JC model contains fewer material constants, which are easy to identify using experimental data. In addition, it has been implemented in various finite element simulation packages. It has been employed to introduce the flow behavior of different metals and alloys [11,12,13,14,15]. Despite that, the JC model assumes no coupling effect between strain hardening, strain rate, and softening parameters, which makes the model fail to predict the flow behavior of a number of alloys accurately, especially at severe conditions [12,16,17,18]. Accordingly, many modifications for the JC model were introduced by researchers, which somehow took the coupling effect between strain hardening, strain rate, and softening parameters into account. [14,19,20,21]. He et al. [22] presented a modification of the JC model of the studied X12 alloy at elevated temperatures. A good predictability of the X12 alloy behavior at elevated temperatures was provided when compared to the predictability of the original JC model.

In 1969, Jonas et al. [23] presented their well-known phenomenological model using the Arrhenius-type equation to study material behavior at hot deformation. The relationship between strain rate, temperature, and stress at hot deformation were considered in their model. The Arrhenius model was used to introduce the behavior of different alloys at elevated temperatures [24,25,26]. However, in their model, the effect of strain hardening on the flow behavior of the material was not considered, which makes the model sometimes fail to predict the flow behavior for some alloys accurately. Considering this, Slooff et al. [27] studied the effect of strain on the Arrhenius-type equation parameters and presented a strain-compensated Arrhenius-type model that takes strain hardening into account along with strain rate and softening. The strain-compensated Arrhenius-type model was successfully implemented to study the behavior of different alloys at hot deformation [28,29,30,31].

Dissimilar to the phenomenological models, a mathematical model is not required to represent the flow behavior of materials at elevated temperatures using the Artificial Neural Network (ANN). The ANN is an artificial intelligence approach that can be employed to model the flow behavior of materials without the utilization of traditional numerical methods. It is a robust approach for handling complex and non-linear relationships, such as strain hardening, strain rate, and temperature relationships during hot deformation. Consequently, the ANN has been recently employed for predicting the hot deformation behavior of metals and alloys. Sabokpa et al. [32] predicted the high temperature flow behavior of an AZ81 magnesium alloy using ANN. The results showed that the ANN model can accurately predict the flow behavior of the AZ81 magnesium alloy. Haghdadi et al. [33] developed an ANN model to predict the hot deformation behavior of an A356 aluminum alloy. The results demonstrated that ANN succeeded to predict the flow behavior of A356 aluminum alloy precisely.

In this work, the flow behavior of the X12 alloy at elevated temperatures will be studied and predicted. Based on the literature introduced in this study, the behavior of the hot deformation of the X12 alloy will be established using the strain-compensated Arrhenius-type model, in addition to two ANN models. The results will be then compared to the modified JC model that was recently presented for the X12 alloy in [22]. Furthermore, the predictability of the four models will be assessed using the correlation coefficient (R), Root Mean Squared Error (RMSE), Average Absolute Relative Error (AARE), and Relative Error (RE).

## 2. Experimental Procedure

He et al. [22] studied the hot deformation behavior of the X12 alloy and presented a modified JC model for predicting the flow stress behavior of the X12 alloy at elevated temperatures. Uniaxial hot deformation tests were performed at a combination of temperatures of 950 °C, 1050 °C, 1150 °C, and 1250 °C and strain rates 0.001 s${}^{-1}$, 0.01 s${}^{-1}$, and 0.1 s${}^{-1}$. The tests were conducted using the Gleeble-3800 thermo-mechanical simulator (Dynamic Systems Inc., New York, NY, USA). The samples were heated to 1250 °C and then cooled to the specified temperature. Argon was used to prevent oxidation of the samples’ surface. The chemical compositions of the X12 alloy as presented in [22] are C 0.11–0.33, Si ≤ 0.12, Mn 0.4–0.5, Cr 10.2–10.8, Mo 1.0–1.1, Ni 0.7–0.8, V 0.15–0.25, S ≤ 0.005, P ≤ 0.012, W 0.95–1.05, Al ≤ 0.01, N 0.045–0.06, and Nb 0.04–0.06. In this study, the same material with the same conditions will be utilized to establish the relation between stress, strain, strain rate, and temperature using the strain-compensated Arrhenius-type equation and two different ANN models.

The experimental datasets introduced in [22] were extracted using the open source Plot Digitizer 2.6.3 software. A statistical-based study was conducted to identify the data extraction accuracy of the software utilized. The data chart with the highest non-linearity was chosen and scanned. The datasets were then extracted three times using the Plot Digitizer software. The statistical analysis showed a maximum Mean Squared Error (MSE) of 0.001 for strain values and 0.1254 MPa for stress values. The maximum error percentage was 3.5% for strain values and 0.5% for stress values, given that the percentage error would significantly reduce to less than 1% if the first 10 datasets were excluded. This was due to the fact that the strain values of the very first 10 datasets were very small in comparison to the rest of the data.

## 3. Constitutive Models

### 3.1. Modified JC Model

A modification of the original JC model for the X12 alloy at elevated temperatures was recently presented by He et al. [22]. The modified JC model is given as:

(1) $$\sigma =A_{1}\varepsilon^{n_{1}}\left(1+\left(b_{1}+b_{1}\;\varepsilon +b_{2}\;\varepsilon^{2}\right)\;\ln\varepsilon^{.*}\right)\times \exp\left[\left(\lambda_{1}+\lambda_{2}\;\varepsilon \right)T^{*}\right]$$

where $A_{1}$, $n_{1}$, $b_{1}$, $b_{2}$, $b_{3}$, $\lambda_{1}$, and $\lambda_{2}$ are material constants. $\varepsilon^{.*}$ represents the dimensionless strain rate as is given by the original JC model, i.e., $\varepsilon^{.*}=\varepsilon^{.}/\varepsilon_{0}^{.}$, where $\varepsilon^{.}$ is the strain rate and $\varepsilon_{0}^{.}$ is a chosen reference strain rate. A dimensionless temperature, $T^{*}$, as given in the original JC model is given by $T^{*}=\left(T-T_{ref}\right)/\left(T_{melt}-T_{ref}\right)$, where *T*, $T_{melt}$, and $T_{ref}$ represent temperature, melting temperature, and a chosen reference temperature, respectively.

### 3.2. Strain-Compensated Arrhenius Model

The strain-compensated Arrhenius-type model has been widely used to introduce the behavior of metals and alloys at elevated temperatures. At the time of hot deformation and at the same strain, the flow stress, strain rate, and temperature can be correlated and introduced by the Arrhenius-type [34] using Equation (2):

(2) $$\varepsilon^{.}=F\left(\sigma \right)\;\exp\left(-Q/RT\right)$$

where $\varepsilon^{.}$ is the strain rate (s${}^{-1}$), σ is the flow stress (MPa), *Q* is the deformation activation energy (kJ/mol), *T* is the absolute temperature (K), and *R* is the gas constant, 8.314 J/(mol K). In accordance with the stress level, F(σ) can be stated as follows [35]:

(3) $$F\left(\sigma \right)=A_{1}\sigma^{n_{1}}\;\left(\sigma <70\;\mathrm{MPa}\right)$$

(4) $$F\left(\sigma \right)=A_{2}\exp\left(\beta \sigma \right)\;\left(\sigma >100\;\mathrm{MPa}\right)$$

(5) $$F\left(\sigma \right)=A{\left[\sinh\left(\alpha \sigma \right)\right]}^{n}\;\left(\mathrm{for all}\;\sigma \right)$$

where $A_{1}$, $n_{1}$, $A_{2}$, β, *A*, *n*, and α are material constants, and $\alpha =\beta /n_{1}$. Substituting Equation (5) into Equation (2), the strain rate can be expressed as:

(6) $$\varepsilon^{.}=A{\left[\sinh\left(\alpha \sigma \right)\right]}^{n}\;\exp\left(-Q/RT\right)$$

The Zener–Hollomon parameter (*Z*) can be implemented to introduce the effect of strain rate and temperature on the deformation behavior of the material as follows [36]:

(7) $$Z=\varepsilon^{.}\;\exp\left(Q/RT\right)=A{\left[\sinh\left(\alpha \sigma \right)\right]}^{n}$$

Finally, the flow stress can be expressed as a function of the Zener–Hollomon parameter as follows [37]:

(8) $$\sigma =\frac{1}{\alpha }\left[{\left(\frac{Z}{A}\right)}^{\frac{1}{n}}+{\left({\left(\frac{Z}{A}\right)}^{\frac{2}{n}}+1\right)}^{\frac{1}{2}}\right]$$

### 3.3. Artificial Neural Network Models

**Figure 1:**
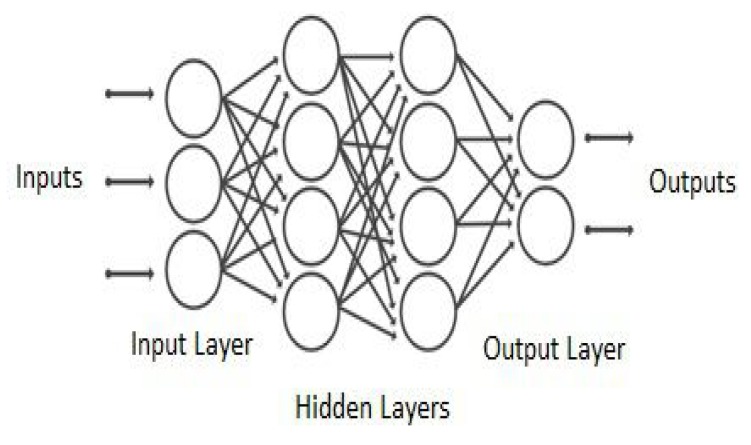
Typical neural network architecture [40].

Artificial neural networks are one of the most popular tools in artificial intelligence. The main element of the ANN is the neuron, and neurons are connected to each other by weighting. The weight of each connection between two neurons can be dynamically adjusted until the target output is achieved. A Multilayer Perceptron (MLP) is a major class of feedforward ANN, and it is commonly used for pattern recognition or function approximation (regression). An MLP is formed of, at least, three layers of neurons and utilizes a nonlinear activation function. These three layers are (a) an input layer, (b) a hidden layer(s), and (c) an output layer. The sigmoidal and hyperbolic tangent functions are the most common activation functions. The basic structure of the MLP is shown in Figure 1. Backpropagation is a supervised learning technique and is used to train MLP networks. The multilayer option and the nonlinear activation function are the features that distinguish the MLP from linear perceptron networks [38,39,40,41].

There are various types of ANN learning algorithms in the literature. However, it is very difficult to know which of these training algorithms will be the most accurate and the fastest for a given problem. Based on a number of experiments conducted using MATLAB (v9.4), the LM algorithm was one of the fastest ANN training algorithms and was able to obtain the lowest MSE when used for regression. Furthermore, the SCG algorithm performed well over a wide variety of regression problems, and hence, it possesses the potential of providing accurate prediction results [40,42]. The LM algorithm is defined as an iterative technique that locates a local minimum of a multivariate function. This function is expressed as the sum of squares of several non-linear and real-valued functions, and it updates its weight and bias values according to the Levenberg–Marquardt technique. The SCG is similar to LM, but it updates its weight and bias values according to the scaled conjugate gradient method. Therefore, this study will employ LM and SCG for the training for the proposed ANN. The Neural Network (NN) was expected to predict the stress values at different strain rates, stain values, and temperature values. Thus, the inputs were the strain rate, strain value, and temperature value, and the output was the stress value at these conditions.

According to the reviewed ANN models, the most popular and well-proven ANN architecture, training algorithm, activation function, and error calculation method utilized for pattern recognition were employed to carry out this comparison. The Multi-Layer Perceptron (MLP) architecture was utilized in combination with the Scaled Conjugate Gradient-based (SCG) and Levenberg–Marquardt (LM) supervised learning algorithms, in addition to the sigmoid activation function for the hidden layers and the linear activation function for the output layer. The performance of the network was evaluated using the minimum error, maximum error, Mean Absolute Error (MAE), ad Root Mean Squared Error (RMSE) quantitative measure at different neurons and hidden layer configurations [38,39].

## 4. Results and Discussion

### 4.1. Flow Stress Behavior

**Figure 2:**
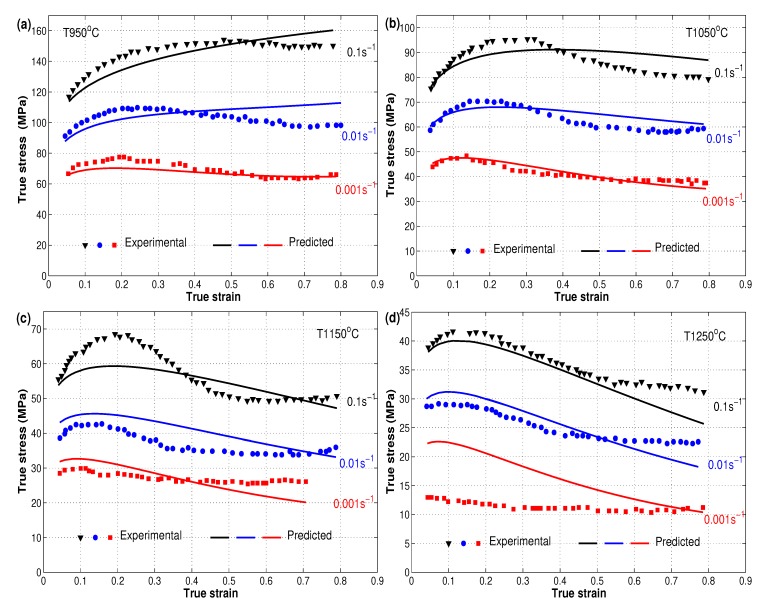
Comparison of experimental stresses (markers) and predicted stresses (lines) by the modified JC model at different strain rates and different temperatures: (**a**) T 950 °C, (**b**) T 1050 °C, (**c**) T 1150 °C, and (**d**) T 1250 °C. Experimental data were taken from [22].

The flow behavior of the X12 alloy at elevated temperatures was presented in reference [22]. The effect of the well-known three phenomena, strain hardening, strain rate, and softening, explain the flow behavior of the hot deformation of the X12 alloy. Due to strain hardening, the flow stress increased quickly from the beginning until the saturation stress with the increase of strain. The effect of strain rate can be noticed with the increase of flow stresses as the strain rate increases at the same temperature, which might be concluded from the generation and multiplication of dislocations. On the other hand, the flow stress decreased to a steady state stress with the high increase of strain due to softening. In addition to that, the value of flow stress decreased as the temperature increased at the same strain rate, which might point to the slow rate of both dynamic recovery and dynamic recrystallization at low temperatures. The increased rate of dynamic recrystallization at high temperatures led to the elimination of dislocation; see Figure 2 for experimental true stress versus true strain data that are presented in markers.

### 4.2. Modified JC Model

The reference strain rate and reference temperature were chosen to be 950 °C and 0.1 s${}^{-1}$ as given in reference [22]. By employing regression analysis for the obtained experimental stresses and strains at different strain rates and temperatures, the material constants were determined using the proposed model. Table 1 represents the values of the constants of the modified JC model for the X12 alloy, as obtained by [22].

The final form of the modified JC model can be given as:

(9) $$\sigma =165.57\varepsilon^{0.13}\left(1+\left(0.0852+0.1042\;\varepsilon -0.0609\;\varepsilon^{2}\right)\;\ln\varepsilon^{.*}\right)\times \exp\left[\left(-1.9365-1.9648\;\varepsilon \right)T^{*}\right]$$

A comparison between experimental stresses and predicted stresses obtained using the modified JC model for the X12 alloy at elevated temperatures is shown in Figure 2 as presented by [22], which is represented again here for comparison reasons with the Arrhenius-type and the two ANN models. The figure shows that the modified JC model gave good predictions of stresses, but a complete behavior of stresses could not be guaranteed. Complex and high non-linearity of the behavior of the X12 alloy at elevated temperatures and different strain rates might be one of the reasons, since the constants of the modified JC model were obtained using a linear regression method.

### 4.3. Strain-Compensated Arrhenius Model

The experimental stress strain data at different strain rates and different temperature values were employed to get the material constants α, *A*, *n*, and *Q*. The determination of these four material constants requires the utilization of different strain values. Therefore, the values of the four material constants were determined at different strain values, i.e., from 0.05–0.8 with an increment of 0.05. In the following, the four material constants were determined at a strain rate of 0.6, and the determination of the four constants at the rest of the strain values followed the same procedure.

To get constants $n_{1}$ and β, which leads to α, substitute Equations (3) and (4) into Equation (2), and considering deformation temperature to be independent, the following relationships are given:

(10) $$\varepsilon^{.}=A_{3}\sigma^{n_{1}}$$

(11) $$\varepsilon^{.}=A_{4}\exp\left(\beta \sigma \right)$$

where $A_{3}$ and $A_{4}$ are material constants. After that, by taking the logarithms for the two sides, the next two equations are obtained:

(12) $$\ln\sigma =\frac{1}{n_{1}}\ln\varepsilon^{.}-\frac{1}{n_{1}}\ln A_{3}$$

(13) $$\sigma =\frac{1}{\beta }\ln\varepsilon^{.}-\frac{1}{\beta }\ln A_{4}$$

**Figure 3:**
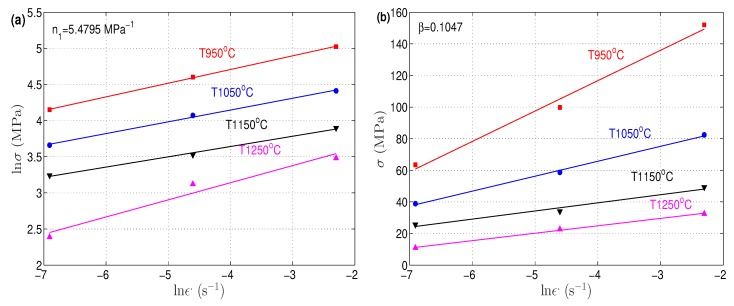
Linear fitting between (**a**) lnσ and $\ln\varepsilon^{.}$ and (**b**) σ and $\ln\varepsilon^{.}$.

At a strain of 0.6, the corresponding stress and strain rate values at different temperatures are plotted (lnσ versus $\ln\varepsilon^{.}$ and σ versus $\ln\varepsilon^{.}$) as shown in Figure 3. The values of $n_{1}$ and β can be determined as the mean reciprocals of the slopes of the lines, which were determined as 5.4795 MPa${}^{-1}$ and 0.1047, respectively. As a consequence, the value of α was equal to 0.0191 $\left(\alpha =\beta /n_{1}\right)$.

To determine constant *n*, the logarithms for both sides of Equation (6) were taken. Assuming *Q* is independent of *T*, the next relation is found:

(14) $$\ln\left[\sinh\left(\alpha \sigma \right)\right]=\frac{1}{n}\ln\varepsilon^{.}+\frac{Q}{nR}\frac{1}{T}-\frac{1}{n}\ln A$$

**Figure 4:**
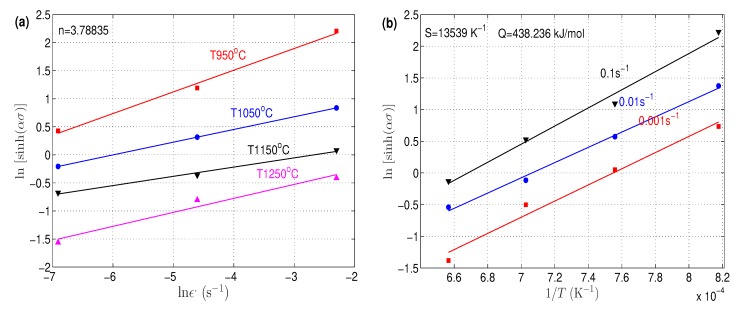
Linear fitting between (**a**) $\ln\left[\sinh\left(\alpha \sigma \right)\right]$ and $\ln\varepsilon^{.}$ and (**b**) $\ln\left[\sinh\left(\alpha \sigma \right)\right]$ and 1/T.

By plotting $\ln\left[\sinh\left(\alpha \sigma \right)\right]$ versus $\ln\varepsilon^{.}$, the value of *n* can be determined as the mean of reciprocals of slopes of the lines, which was determined as 3.78835, as shown in Figure 4a. By taking the derivative of Equation (14) and considering $\ln\varepsilon^{.}$ and 1/T as two independent variables, *Q* can be expressed as:

(15) $$Q=R{\left[\frac{\partial \ln\varepsilon^{.}}{\partial \ln\left[\sinh\left(\alpha \sigma \right)\right]}\right]}_{T}{\left[\frac{\partial \ln\left[\sinh\left(\alpha \sigma \right)\right]}{\partial \left(1/T\right)}\right]}_{\varepsilon^{.}}=RnS$$

where *S* is a constant that can be computed by plotting $\ln\left[\sinh\left(\alpha \sigma \right)\right]$ versus 1/T, and the *S* value was computed as the mean of slopes of lines, which was given as 13,539, as shown in Figure 4b. By substituting into Equation (15), *Q* was computed as 438.236 kJ/mol.

By taking the logarithms for both sides of Equation (7), the next relation is introduced:

(16) $$\ln Z=\ln\left[\sinh\left(\alpha \sigma \right)\right]+\ln A$$

**Figure 5:**
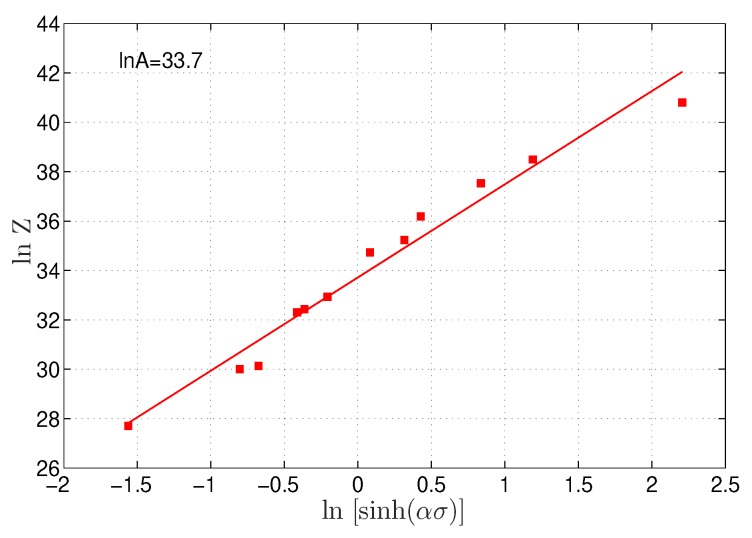
Linear fitting between $\ln\left[\sinh\left(\alpha \sigma \right)\right]$ and lnZ.

lnA can be determined as the mean of intercepts of lines when lnZ is plotted versus $\ln\left[\sinh\left(\alpha \sigma \right)\right]$, as shown in Figure 5, which gave a value of 4.3224 × 10^14^ s${}^{-1}$ for *A*.

**Figure 6:**
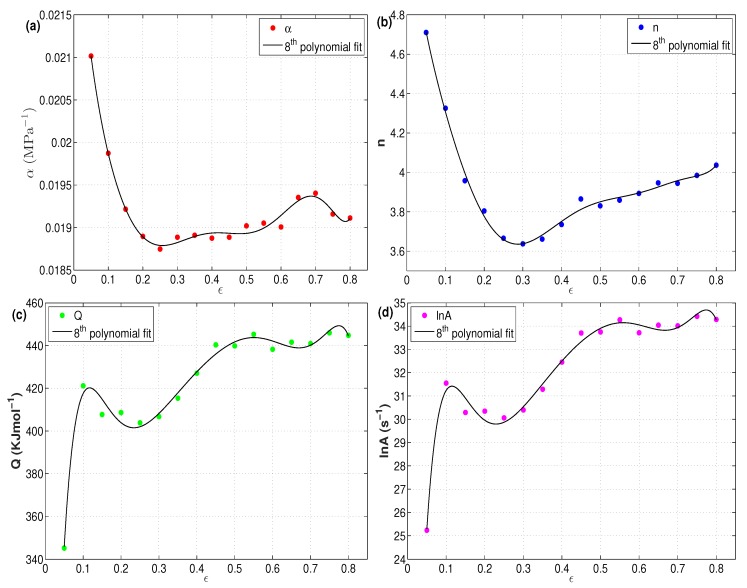
Relationship between strain and constant (**a**) α, (**b**) *n* (**c**) *Q* and (**d**) lnA.

The flow behavior of the X12 alloy at elevated temperature can be predicted by discovering the constants α, *A*, *n*, and *Q* as functions of strain. The relation between the four constants and the strain is shown in Figure 6. The strain value ranged from 0.05–0.8, with an increment of 0.05. The figure shows that the strain had a strong outcome on the resulting four constants. An eighth order polynomial function was established to represent the relation between the strain and the four constants with a high correlation.

**Figure 7:**
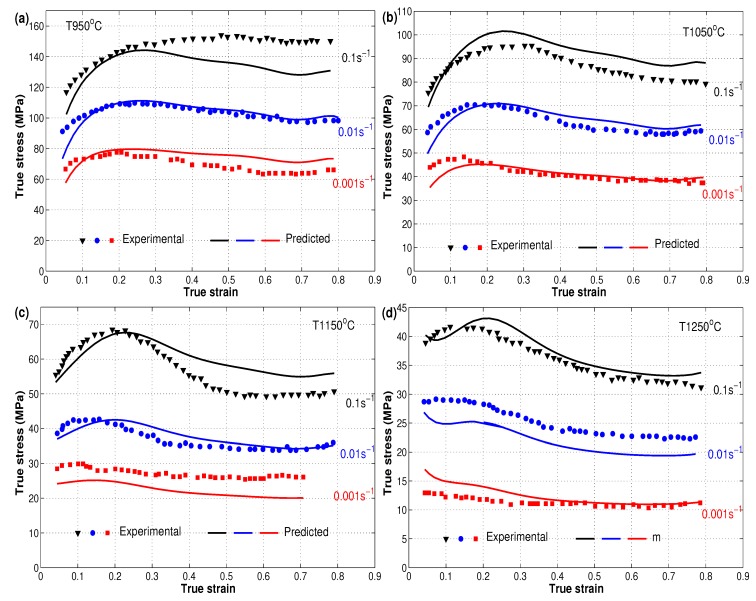
Comparison of experimental stresses (markers) and predicted stresses (lines) by the strain-compensated Arrhenius model at different strain rates and different temperatures: (**a**) T 950 °C, (**b**) T 1050 °C, (**c**) T 1150 °C, and (**d**) T 1250 °C. Experimental data were taken from [22].

By substituting constants α, *A*, *n*, and *Q* as functions of strain with the established eighth polynomial order into Equation (8), the flow behavior of the X12 alloy can be predicted through different strains and with different combinations of strain rates and temperatures. Figure 7 shows the experimental stresses against the predicted stresses obtained by the strain-compensated Arrhenius model for the hot deformation of the X12 alloy. As can be seen, the predicted stresses obtained by the strain-compensated Arrhenius model were in good agreement with the experimental stresses of the X12 alloy. However, the model failed to predict stresses at some combinations of strain rates and temperatures accurately, such as 0.1 s${}^{-1}$ and T 950 °C and 0.01 s${}^{-1}$ and T 1250 °C. One possible reason is the high non-linearity of the hot deformation behavior of the X12 alloy. Another suggested reason is the high dependency of the parameters α, *A*, *n*, and *Q* on the strain, which might require a large selected range of values of strain to fit these parameters, which might be considered as costly in terms of time.

### 4.4. Artificial Neural Network Models

**Figure 8:**
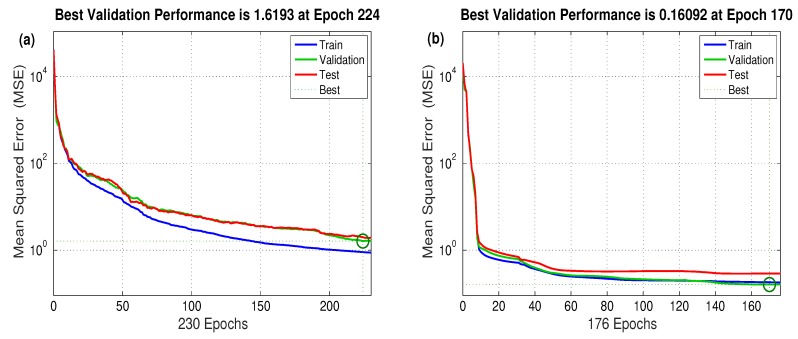
Validation performances of the LM and the SCG-trained ANN networks: (**a**) the SCG-trained network and (**b**) the LM-trained ANN network.

The assessment of each NN performance incorporated 100 neural network configurations with single and double hidden layers; each hidden layer contained a number of neurons ranging from 1 neuron–50 neurons. In this study, 482 datasets were chosen from the flow stresses along the whole strain interval. The ANN was trained using 70% of the total number of datasets and was validated and tested using the remaining datasets. Hence, 338 datasets were utilized for training and 144 datasets for validation and testing. Among the 100 trials, the performance of the SCG-trained ANN network, shown in Figure 8a, was the best for a two-layer network configuration with 17 neurons per each layer. For the LM-trained ANN network, the performance, shown in Figure 8b, was the best for a single-layer configuration with a total of 18 neurons. It was obvious that the LM-trained one had a better performance with a Mean Squared Error (MSE) of as low as 0.16092.

Table 2 shows a quantitative comparison between the two best ANN configurations. The results showed that the ANN network trained using LM technique had a better performance with an MAE and RMSE of 0.374 MPa and 0.474 MPa, respectively. However, the results obtained using the ANN network that was trained using the SCG technique were still far better than the statistical based model results with an MAE and RMSE of 0.811 MPa and 1.082 MPa, respectively. The average computational time needed for the prediction of a stress value using the LM approach was 249 milliseconds using an OPTIPLEX 7440 machine (Dell Company, Round Rock, TX, USA) with Intel Core(TM) I7-6700 CPU @ 3.4-GHz processor.

**Figure 9:**
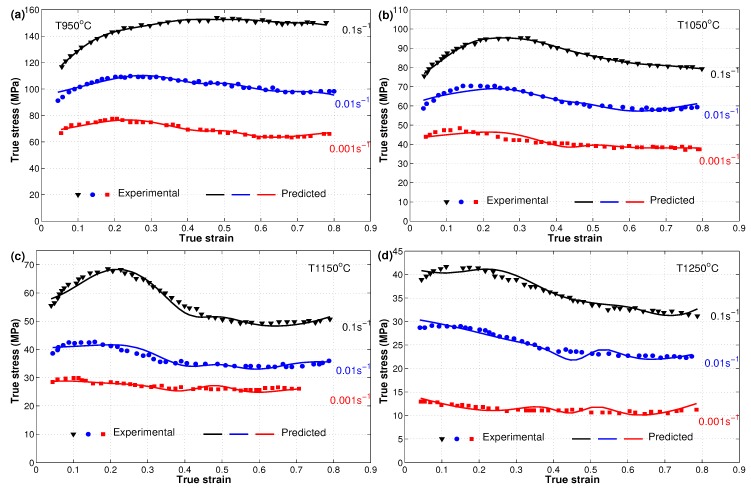
Comparison of experimental stresses (markers) and predicted stresses (lines) by the developed ANN model using the SCG technique at different strain rates and different temperatures (**a**) T 950 °C, (**b**) T 1050 °C, (**c**) T 1150 °C, and (**d**) T 1250 °C. Experimental data were taken from [22].

**Figure 10:**
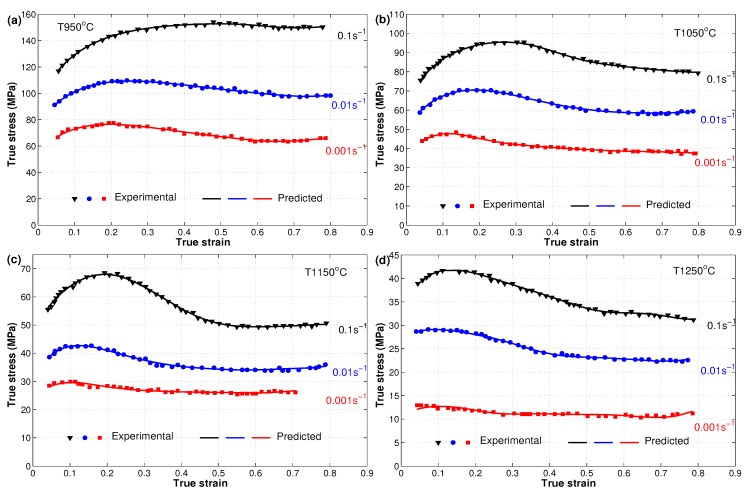
Comparison of experimental stresses (markers) and predicted stresses (lines) by the developed ANN model using the LM technique at different strain rates and different temperatures: (**a**) T 950 °C, (**b**) T 1050 °C, (**c**) T 1150 °C, and (**d**) T 1250 °C. Experimental data were taken from [22].

The predicted stresses obtained by the developed ANN using both the SCG and LM techniques were compared to experimental stresses for the hot deformation of the X12 alloy, as shown in Figure 9 and Figure 10. Clearly, the developed ANN models succeeded to predict the flow behavior of the X12 alloy at elevated temperatures with high accuracy. The figures show that the developed ANN model using the SCG and LM training technique managed to predict the flow behavior of the X12 alloy precisely. It is also obvious that the LM-trained model had a better prediction accuracy when compared to the SCG-trained model. The high quality of dealing with steep non-linear systems, i.e., at high temperatures and different strain rates, might be the reason that the ANN models delivered extraordinary predictions.

### 4.5. Comparative Study between the Modified JC, Strain-Compensated Arrhenius, and Developed ANN Models

**Figure 11:**
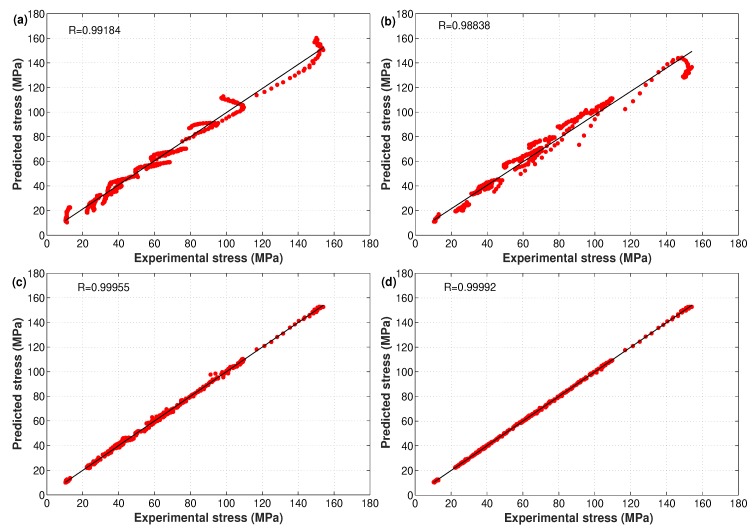
Correlation of experimental and predicted stresses obtained by (**a**) the modified JC model, (**b**) the strain-compensated Arrhenius model, (**c**) the developed ANN model using SCG, and (**d**) the developed ANN model using LM. Experimental data were taken from [22].

As can be observed from Figure 2, Figure 7, Figure 9, and Figure 10, both developed ANN models fit the experimental stresses very well. Furthermore, it can be seen that the developed ANN using the LM approach was more appropriate as it could predict the flow behavior of the X12 alloy with more precision. In order to investigate the predictability of the modified JC, strain- compensated Arrhenius, and developed ANN models, a comparative analysis using the standard statistical parameters R and AARE [43,44] was concluded and computed as follows:

(17) $$R=\frac{\sum_{i=1}^{N}\left(\sigma_{exp}-\bar{\sigma_{exp}}\right)\;\left(\sigma_{pred}-\bar{\sigma_{pred}}\right)}{\sqrt{\sum_{i=1}^{N}{\left(\sigma_{exp}-\bar{\sigma_{exp}}\right)}^{2}\;\sum_{i=1}^{N}{\left(\sigma_{pred}-\bar{\sigma_{pred}}\right)}^{2}}}$$

(18) $$\mathrm{AARE}=\frac{1}{N}\sum_{i=1}^{N}\left|\frac{\sigma_{exp}-\sigma_{pred}}{\sigma_{exp}}\right|\times 100$$

where $\sigma_{exp}$ represents experimental stresses, $\sigma_{pred}$ gives predicted stresses at the determined constants in the constitutive models, $\bar{\sigma_{exp}}$ is the mean value of $\sigma_{exp}$, $\bar{\sigma_{pred}}$ is the mean value of $\sigma_{pred}$, and *N* is the total number of observations. Figure 11 shows the correlation between experimental stresses and predicted stresses for the hot deformation of the X12 alloy obtained by the modified JC, strain-compensated Arrhenius, and developed ANN models. Obviously, the developed ANN model using the LM approach had the highest R value (0.99992) when compared to the R value of the modified JC model (0.99184), the R value of the strain-compensated Arrhenius model (0.98838), and the R value of the SCG-trained ANN model (0.99955).

In fact, the correlation coefficient, R, supplied information of the power of the linear relationship between experimental and predicted data. Sometimes, it tended to higher or lower values. Therefore, a better performance cannot always be considered with high R values, which is well known as a biased statistical parameter [45]. Consequently, the unbiased statistical parameter AARE (see Equation (18)) that computes the relative error for each term, might be considered as a good parameter that ensures better performance [46]. In addition, the RMSE will be considered as it was used for the verification of the modified JC model for the X12 alloy at elevated temperatures by He et al. [22] as follows:

(19) $$\mathrm{RMSE}=\frac{1}{N}\sqrt{\sum_{i=1}^{N}{\left(\sigma_{exp}-\sigma_{pred}\right)}^{2}}$$

A comparison between R, AARE, and RMSE of experimental stresses and predicted stresses for the hot deformation behavior of the X12 alloy obtained by the modified JC, strain-compensated Arrhenius, and proposed ANN models is shown in Table 3. As can be observed from the table, the developed ANN model using the LM approach gave the highest R and lowest AARE and RMSE values, which confirmed the high precision prediction capability of the proposed model. The modified JC model gave high R values and lower AARE and RMSE values when compared to the strain-compensated Arrhenius model, with no big differences. The accuracy of prediction was far less than the one obtained using ANN, which made both models non-favorable for the prediction of the flow behavior of the X12 alloy at elevated temperatures. Once again, the complex behavior and high non-linearity of the hot deformation of the X12 alloy might be a reason for the superior performance of the developed ANN models.

In addition to the statistical parameters, R, AARE, and RMSE, the Relative Error (RE) between experimental and predicted stresses for each and every stress was computed, for further investigation as a comparison tool between the modified JC, strain-compensated Arrhenius, and the developed ANN models. Equation (20) is utilized for the calculation of RE percentage:

(20) $$\mathrm{RE}=\left(\frac{\sigma_{exp}-\sigma_{pred}}{\sigma_{exp}}\right)\times 100$$

**Figure 12:**
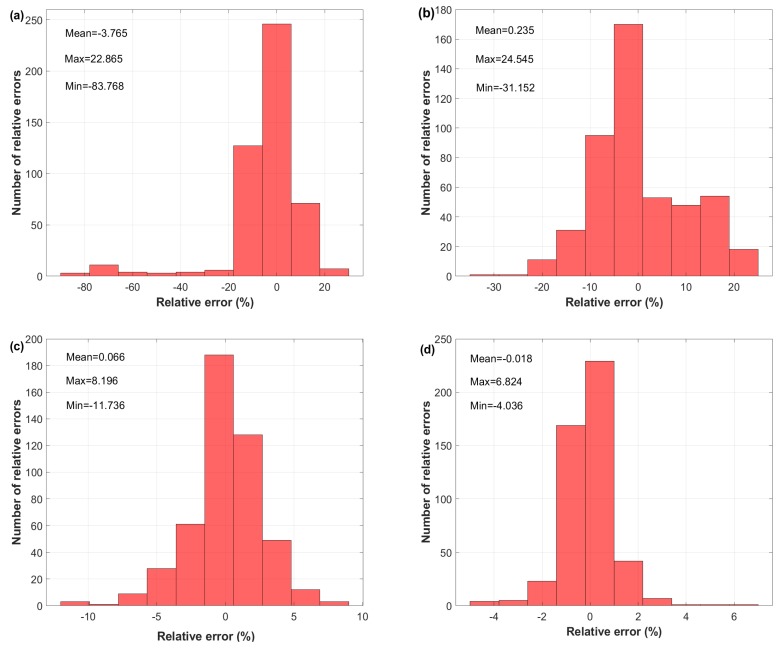
Histogram of the relative error obtained by (**a**) the modified JC model, (**b**) strain-compensated Arrhenius model, (**c**) developed ANN model using SCG, and (**d**) developed ANN model using LM.

Figure 12 shows a histogram of the relative error for the modified JC, strain-compensated Arrhenius, and the developed ANN models for the hot deformation of the X12 alloy. Among the RE values of the considered models, the figure shows that the developed ANN model using the LM technique had the lowest RE values, with a minimum to maximum RE value −4.036–6.824% and with a mean RE value −0.018%. The computed statistics of the RE revealed the better performance of the proposed ANN models, with a clearly better performance of the LM-trained ANN than the SCG-trained one. In the meantime, 99.6% and 92.74% of the numbers of relative errors of the developed ANN model using the LM and SCG training algorithms were approximately limited between RE values of −5% and 5%. In comparison to the ANN models, a large percentage of RE numbers for both modified JC and strain-compensated Arrhenius models were obtained, 42.59% and 43.78% respectively, for the same range of RE values, i.e., −5–5%. In sum, the relative error results confirmed that the developed ANN models gave more accurate results than the modified JC and strain-compensated Arrhenius models and were in agreement with the statistical results of R, AARE, and RMSE.

## 5. Conclusions

The flow behavior of the X12 alloy at elevated temperatures was studied. In order to predict the flow stress behavior of the X12 alloy accurately, a comparative study between a modified JC model, which was recently published about the aforementioned alloy, a strain-compensated Arrhenius model, in addition to two ANN models was carried out.

The modified JC and strain-compensated models could be utilized to predict the hot deformation behavior of the X12 alloy with a good accuracy, but only for some of the tested domain of temperatures and strain rates, with R values of 0.992 and 0.988 respectively, and with AARE and RMSE values of 9.367% and 4.690 MPa for the modified JC model and of 7.557% and 5.531 MPa for the strain-compensated model, respectively.

However, the predictions of the phenomenological models were not accurate enough and did not provide a precise prediction for the flow stress. Hence, two ANN-based models were developed with a view toward improving the prediction accuracy. The ANN-based models yielded a superior prediction accuracy, in particular the LM-trained ANN model with R, AARE, and RMSE values of 0.99992, 0.748%, and 0.441 MPa, respectively. Regarding the RE values, among the four proposed models, the LM-trained ANN model produced the best relative error results when compared to the other three models.

As a matter of fact, strain, strain rate, and temperatures are non-linear parameters, and thus, the flow behavior of the X12 alloy at elevated temperatures is highly non-linear and of high complexity. This is why the modified JC and strain-compensated Arrhenius models could not accurately predict the flow behavior of the hot deformation of the X12 alloy, as all constants in both models were determined using the linear regression method. This justifies the superiority of the ANN results over the other addressed methods. As a result, the development of such an ANN model will help the designers and researchers to predict the behavior of this alloy at high-temperature values accurately.

## Figures and Tables

**Table 1:** Material constants of the modified Johnson–Cook (JC) model for hot deformation of the X12 alloy obtained by [22].

Material Constant	$A_{1}$ (MPa)	$n_{1}$	$b_{1}$	$b_{2}$	$b_{3}$	$\lambda_{1}$	$\lambda_{2}$
Value	165.57	0.13	0.0852	0.1042	−0.0609	−1.9365	−1.9648

**Table 2:** Quantitative comparison of the best performing ANN networks.

Assessment Method	17 × 17 Neurons NN Using SCG		18 Neurons NN Using LM	
	All Data	Testing Data	All Data	Testing Data
Min. error	−3.013	−3.013	−1.800	−1.800
Max. error	6.417	6.417	1.294	1.231
MAE	0.811	1.010	0.334	0.374
RMSE	1.082	1.344	0.441	0.474

**Table 3:** R, Average Absolute Relative Error (AARE), and RMSE of the modified JC, strain-compensated Arrhenius, and developed ANN models. SCG, Scaled Conjugate Gradient.

Model	R	AARE (%)	RMSE (MPa)
Modified JC	0.99184	9.367	4.690
Strain compensated Arrhenius	0.98838	7.557	5.531
Developed ANN using SCG	0.99955	1.908	1.082
Developed ANN using LM	0.99992	0.748	0.441
